# *Mycobacterium tuberculosis* 10-kDa co-chaperonin regulates the expression levels of receptor activator of nuclear factor-κB ligand and osteoprotegerin in human osteoblasts

**DOI:** 10.3892/etm.2014.2153

**Published:** 2014-12-22

**Authors:** YUANYU ZHANG, XIA LIU, KUN LI, JINGPING BAI

**Affiliations:** 1 Department of Orthopedics, Affiliated Tumor Hospital of Xinjiang Medical University, Urumqi, Xinjiang 830000, P.R. China; 2 Department of Pathology, First Affiliated Hospital of Xinjiang Medical University, Urumqi, Xinjiang 830000, P.R. China

**Keywords:** recombinant *Mycobacterium tuberculosis* 10-kDa co-chaperonin, osteoprotegerin, bone resorption, nuclear factor-κB ligand, osteoblast, bone tuberculosis

## Abstract

The aim of the present study was to investigate the effect of recombinant *Mycobacterium tuberculosis* (r-Mt) 10-kDa co-chaperonin (cpn10) on the expression of osteoprotegerin (OPG) and receptor activator of nuclear factor-κB ligand (RANKL) in third-generation cultured osteoblasts. The osteoblast-like cultures were isolated from bone fragments taken from patients undergoing surgery. Prior to stimulation with r-Mt cpn10, cells were incubated in serum-free medium for 24 h. r-Mt cpn10 was added into fresh serum-free medium, reaching final concentrations of 0.01–10 μg/ml. The levels of OPG were determined using enzyme-linked immunosorbent assay. Reverse transcription-quantitative polymerase chain reaction (RT-qPCR) analysis was performed to determine the levels of RANKL and OPG mRNA. For measurement of the protein levels of OPG and RANKL, a western blotting assay was performed. r-Mt cpn10 downregulated the protein levels of OPG in the third generation cultured osteoblasts at a dose of 10 μg/ml. RT-qPCR revealed that the OPG mRNA level was decreased by 73% after 4 h and by 85.5% after 8 h following incubation with r-Mt cpn10 (10 μg/ml). Western blot analysis demonstrated similar results for the OPG protein level. In the third-generation cultured osteoblasts, the levels of RANKL mRNA and protein were increased by 2.6- and 1-fold, respectively, following incubation with r-Mt cpn10 (10 μg/ml). Furthermore, the RANKL/OPG ratio was markedly increased by r-Mt cpn10 (10 μg/ml) treatment. In conclusion, the results of the current study demonstrated that r-Mt cpn10 decreased the levels of OPG and increased the levels of RANKL in a dose- and time-dependent manner. Notably, the present study indicated that r-Mt cpn10 exerts its effect on osteoblastic cells by increasing the RANKL/OPG ratio.

## Introduction

Osteoblasts and osteoclasts maintain normal bone metabolism balance, and are involved in bone metabolism and the immune activity of the body (1). Receptor activator of nuclear factor-κB ligand (RANKL) expression, which can be found on the surface of osteoblasts, is activated by stimulation with endocrine hormones and local cytokines, and can be combined with RANK receptors on the surface of osteoclasts to induce osteoclast proliferation and activation (2).

At present, the known mechanism of osteoclast formation is the RANK-RANKL-osteoprotegerin (OPG) shaft (3–5). RANK lies on the surface of osteoclast precursors. Osteoblasts and bone marrow stromal cells express RANKL to promote osteoclast differentiation, enhance the viability of mature osteoclasts and prevent osteoclast apoptosis. Certain cells, including bone marrow stromal cells and osteoblasts, express OPG, which inhibits the differentiation and maturation of osteoclasts to decrease bone resorption activity and induce apoptosis. Regulators, including serum osteoprotegerin, serve a function in the homeostasis of bone resorption and reconstruction, and the whole process is regulated by osteoblasts (6,7).

Bones and joints are the most commonly affected organs in extrapulmonary tuberculosis. The use of anti-tuberculosis drugs has allowed the control of tuberculosis in the past (8); however, due to human immunodeficiency virus infection/acquired immune deficiency syndrome, drugs, immunosuppressants, alcoholism and poverty, the incidence of tuberculosis is once more on the increase, particularly in developing countries (9). Bone absorption and vertebral body structure damage are pathological changes caused by bone tuberculosis. Pott’s disease (spinal tuberculosis) can cause bone destruction, spinal cord compression and nerve dysfunction. *In vitro*, recombinant *Mycobacterium tuberculosis* (r-Mt) 10-kDa co-chaperonin (cpn10) causes accelerated bone resorption and fragility (10–12), increases osteoclast proliferation and activity, and stimulates bone resorption, thus inhibiting osteoblast proliferation (13); however, whether the effect of r-Mt cpn10 in stimulating bone resorption is mediated by any receptors expressed in osteoblasts remains unclear. Furthermore, it is not well understood how r-Mt cpn10 affects bone tissue at the molecular level. In the present study, the effect of r-Mt cpn10 on the expression levels of OPG and RANKL in third cultured osteoblasts was investigated.

## Materials and methods

### Reagents

Cell culture media, penicillin (100 U/ml), streptomycin (100 U/ml), L-glutamine, phosphate-buffered saline, trypsin-EDTA, super fetal bovine serum and r-Mt cpn10 were purchased from Shanghai Biological Engineering Co., Ltd. (batch no. 120628; Shanghai, China). Freeze-dried r-Mt cpn10 protein was stored under nitrogen flow and dissolved in double-distilled water prior to use.

### Cell culture

The isolation and culture of the third-generation osteoblasts was performed according to the methods used in a previous study (14). The original osteoblast-like cultures were isolated from bone fragments taken from a total of 12 male patients undergoing surgery, none of which had metabolic bone disease. Written informed consent was obtained from all patients prior to the study. Briefly, the trabecular bone was cut into sections (3–4-mm), thoroughly rinsed and vortexed five times in phosphate-buffered saline at 1,200 × g for 15 min with a Low-Speed Desktop Centrifuge (Lu Xiangyi Centrifuge Instrument Co., Ltd., Shanghai, China). Cells were incubated in a humidified CO_2_ incubator at 37°C and the medium was changed twice a week until confluence was achieved. Cells were cultured in Dulbecco’s Modified Eagle’s Medium supplemented with 10% super fetal bovine serum, L-glutamine (2 mM) and antibiotics. The osteoblastic phenotype of the cells in the third generation osteoblast-like cultures was verified by biochemical markers as previously described (14). Prior to stimulation with r-Mt cpn10, cells were incubated in serum-free medium for 24 h. r-Mt cpn10 was added into fresh serum-free medium, reaching final concentrations of 0.01–10 μg/ml. The controls were incubated with fresh serum-free medium alone. For the measurement of OPG secretion, 500,000 third-generation human osteoblasts were seeded in triplicate into 25-cm^2^ dishes. After 24 h, aliquots of the medium were collected and the number of cells in each well was manually counted. For RNA isolation, cells were seeded in 25-cm^2^ flasks at a density of 150,000 cells/flask. Cultures were harvested 2, 4, 8 and 24 h after stimulation with r-Mt cpn10. For protein isolation, cells were seeded in 25-cm^2^ flasks at a density of 600,000 cells/flask. Cultures were harvested 2, 4, 8 and 24 h after stimulation with r-Mt cpn10. The present study was approved by the Ethics Committee of the People’s Hospital of Xinjiang Autonomous Region (registration no. 2012084; Urumqi, China).

### Enzyme-linked immunosorbent assay (ELISA)

The levels of OPG were determined using ELISA as described previously (15). Briefly, a MaxiSorp™ microtiter plate (Nalge Nunc International, Penfield, NY, USA) was coated with mouse anti-human OPG monoclonal antibody (cat. no. ab2147; dilution, 1:50; Abcam, Cambridge, MA, USA). Samples or standard protein recombinant human OPG were added and incubated for 2 h at room temperature. The bound proteins were detected with biotinylated goat anti-human OPG antibody (batch no. 40849; Shanghai YongYe Biotechnology Co., Ltd., Shanghai, China). Following development, the plate was read at 450 nm using a microplate reader (MF Benchmark Plus reader; Bio-Rad, Hercules, CA, USA). The detection limit was 35 pg/ml, and the intra- and inter-assay variations were 6.2 and 21%, respectively. Protein concentrations were normalized to the number of cells and expressed as pg/ml/10^6^ cells.

### Reverse transcription-quantitative polymerase chain reaction (RT-qPCR)

RT-qPCR analysis was performed in triplicate for the determination of the levels of RANKL and OPG in the third-generation osteoblasts. To isolate the total RNA, TRIzol^®^ (Invitrogen Life Technologies, Carlsbad, CA, USA) and SYBR^®^ Select Master Mix (Applied Biosystems^®^; Invitrogen Life Technologies) were used according to the manufacturers’ instructions. RNA quality was analyzed using a 2100 series Bioanalyzer Instrument (Agilent Technologies, Santa Clara, CA, USA). RNA concentration and purity were measured by determining the 260/280 nm ratio. All ratios were >1.8. RNA (1 μg/sample) were reverse-transcribed to first-strand cDNA with a RevertAid™ First Strand cDNA Synthesis kit (Thermo Fisher Scientific, Inc., Waltham, MA, USA) and used in RT-qPCR analysis in an ABI^®^ 7500 Real-Time PCR system (Applied Biosystems; Invitrogen Life Technologies). All RNA samples from the same experiment were transcribed at the same time and each cDNA was analyzed in duplicate. Fluorescent fluorescein amidite-labeled probes and gene-specific primers spanning over the exon-intron boundary for RANKL and OPG were used (Shanghai Biological Engineering Co., Ltd.). A 4,5-dichloro-2,7-dimethoxy-fluorescein-labeled reference gene, GAPDH, was used as an endogenous control. Standard curves for various genes were achieved through a 1×10^−2^-1×10^−8^ dilution series of a verified PCR product with a concentration of 10 ng/μl. The amplification and analysis of cDNA fragments were performed on a thermocycler (iCycler; Bio-Rad, Munich, Germany). The primers used in this section were as follows: GAPDH forward, 5′-TGTTGCCATCAATGACCCCTT-3′ and reverse, 3′-GCGACTCATGCAGCACCTC-5; OPG forward, 5′-CCTCTGTGAAAACAGCGTGC-3′ and reverse, 3′-TTTACCGCTGGTTCTGTGGA-5′; RANKL forward, 5′-GGAGTTGGCCGCAGACAAGA-3′ and reverse, 3′-TCG CAGCGGGACAAGAAGAT-5′.

### Western blotting

To determine the levels of OPG, RANKL and GAPDH, equal quantities of proteins (40 μg) were separated by 10% sodium dodecyl sulfate-polyacrylamide gel electrophoresis and blotted with polyvinylidene fluoride membranes (Millipore, Billerica, MA, USA). Following blocking with 5% skimmed milk and washing with 1X Tris-buffered saline with Tween-20 (20 mM Tris-HCL, pH 7.5; 150 mM NaCl; 0.1% Tween-20; all purchased from Sigma, St. Louis, MO, USA), the membranes were probed with rabbit polyclonal anti-RANKL (dilution, 1:800; cat. no. bs-0747R; Bioss, Beijing, China), rabbit polyclonal anti-OPG (dilution, 1:800; cat. no. bs-0431R; Bioss) at room temperature for 1 h and then incubated with anti-mouse or anti-rabbit immunoglobulin G conjugated with horseradish peroxidase (Zhongshan Jinqiao Biological Technology Co., Ltd., Beijing, China.). Following final treatment with Amersham™ ECL™ Western Blotting Detection reagents (Amersham; GE Healthcare, Little Chalfont, UK), the samples were exposed to X-ray film and relevant protein bands were recorded.

### Statistical analysis

Statistical analysis was performed using SPSS 17.0 software (SPSS, Inc., Chicago, IL, USA). The mRNA levels are expressed as mean arbitrary units ± intra-assay variation of duplicate analyses. Each experiment was repeated for the assessment of inter-assay variation and differed by <10%. For protein production measured with ELISA and western blotting, the statistical significance was calculated from three separate experiments and evaluated by the Student’s t-test. P<0.05 was considered to indicate a statistically significant difference.

## Results

### r-Mt cpn10 downregulates OPG protein secretion

**Figure 1:**
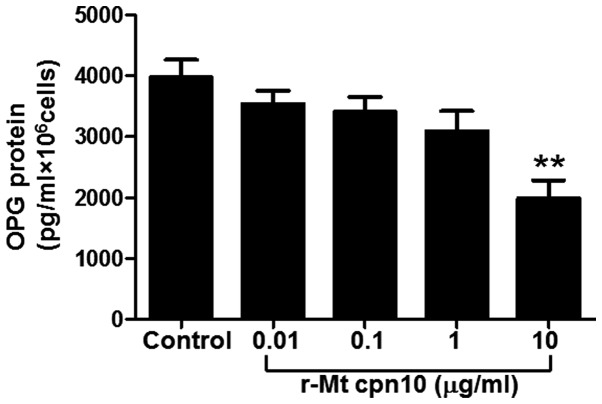
Dose-response effect of r-Mt cpn10 on OPG protein secretion in third-generation cultured osteoblasts. The cells were cultured in serum-free medium for 24 h prior to stimulation with different doses of r-Mt cpn10. After 24 h of stimulation, aliquots of the medium were collected and the concentration of OPG protein was determined with enzyme-linked immunosorbent assay. Protein concentrations were normalized to the number of cells, which was counted manually. Values were obtained from three independent experiments and are expressed as the mean ± standard deviation. ^**^P<0.01 compared with the control. r-Mt, recombinant *Mycobacterium tuberculosis*; cpn10, 10-kDa co-chaperonin; OPG, osteoprotegerin.

To assess the effect of r-Mt cpn10 on OPG protein secretion, the third-generation cultured osteoblasts were treated with r-Mt cpn10 at doses ranging from 0.01 to 10 μg/ml in serum-free media. The results revealed that lower doses (0.01, 0.1 and 1 μg/ml) of r-Mt cpn10 failed to significantly decrease OPG protein secretion; however, 10 μg/ml r-Mt cpn10 significantly decreased OPG protein secretion by 50% compared with the control (P<0.01) (Fig. 1). This result suggested that r-Mt cpn10 downregulated OPG protein secretion at 10 μg/ml.

### r-Mt cpn10 inhibits OPG mRNA and protein expression in a time-dependent manner

**Figure 2:**
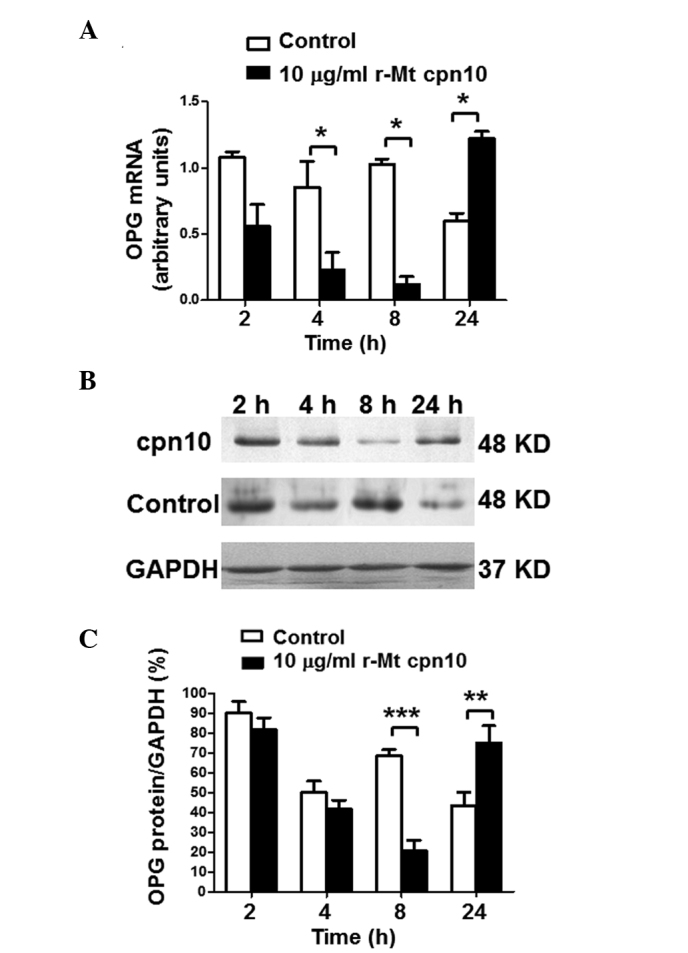
Levels of OPG in third-generation osteoblasts following treatment with r-Mt cpn10 (10 μg/ml) for 2, 4, 8 and 24 h. (A) Levels of OPG mRNA following stimulation with control or r-Mt cpn10 (10 μg/ml) for 2, 4, 8 and 24 h. Total RNA was isolated from the harvested cells, transcribed to cDNA and quantified by reverse transcription-quantitative polymerase chain reaction analysis. The amount of OPG mRNA was compared with the amount of the reference gene (GAPDH) mRNA. Values are expressed as the mean ± intra-assay variation of duplicate analyses. (B) Western blotting of OPG protein expression following treatment with the control or r-Mt cpn10 (10 μg/ml) for 2, 4, 8 and 24 h. (C) Quantification of the level of OPG protein following treatment with the control or r-Mt cpn10 (10 μg/ml) for 2, 4, 8 and 24 h. Results are expressed as the mean ± standard deviation. ^*^P<0.05, ^**^P<0.01 and ^***^P<0.001. r-Mt, recombinant *Mycobacterium tuberculosis*; cpn10, 10-kDa co-chaperonin; OPG, osteoprotegerin.

RT-qPCR and western blotting were performed to investigate the time-course effect of r-Mt cpn10 on the levels of OPG. The RT-qPCR results for the third-generation cultured osteoblasts demonstrated that r-Mt cpn10 (10 μg/ml) treatment decreased OPG mRNA expression to 27% of that of the control after 4 h, and to 14.5% of that of the control after 8 h; however, it increased OPG mRNA expression to 200% of that of the control after 24 h (Fig. 2A). Western blot analysis revealed a similar effect of r-Mt cpn10 (10 μg/ml) on OPG protein expression, with the maximal inhibitory effect occurring after 8 h (Fig. 2B and C). These results indicated that r-Mt cpn10 inhibited OPG mRNA and protein expression in a time-dependent manner.

### r-Mt cpn10 inhibits OPG mRNA and protein expression in a dose-dependent manner

**Figure 3:**
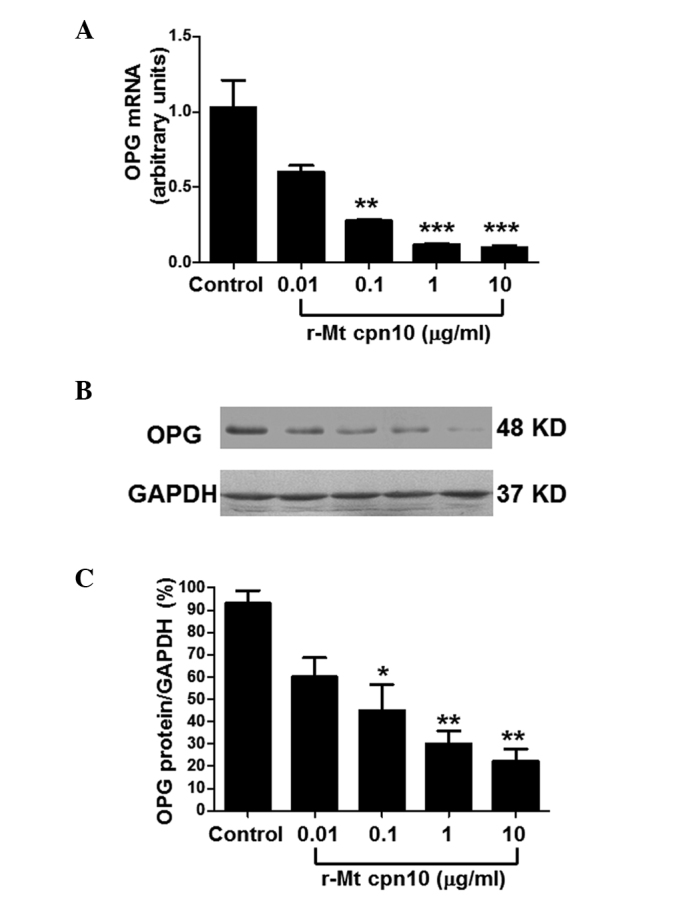
Levels of OPG in third-generation osteoblasts following treatment with different doses of r-Mt cpn10 for 8 h. (A) Levels of OPG mRNA following treatment with the control or different doses of r-Mt cpn10 for 8 h. Cells were grown in serum-free medium for 24 h prior to stimulation. Total RNA was isolated from the harvested cells, transcribed to cDNA, and quantified by reverse transcription-quantitative polymerase chain reaction analysis. The amount of OPG mRNA was compared with the amount of the reference gene (GAPDH) mRNA. Values are expressed as the mean ± intra-assay variation of duplicate analyses. (B) Western blotting of OPG protein expression following treatment with the control or r-Mt cpn10 (0.01, 0.1, 1 and 10 μg/ml) for 8 h. (C) Quantification of the level of OPG protein following treatment with the control or r-Mt cpn10 (0.01, 0.1, 1 and 10 μg/ml) for 8 h. Results are expressed as the mean ± standard deviation. ^**^P<0.01 and ^***^P<0.001 compared with the control. r-Mt, recombinant *Mycobacterium tuberculosis*; cpn10, 10-kDa co-chaperonin; OPG, osteoprotegerin.

RT-qPCR and western blotting were performed to investigate the effect of different doses of r-Mt cpn10 on the levels of OPG. Data from the RT-qPCR revealed that the level of OPG mRNA decreased following treatment with r-Mt cpn10 (0.01, 0.1, 1 and 10 μg/ml) for 8 h. The higher the dose of r-Mt cpn10, the lower the OPG mRNA level (Fig. 3A). The level of OPG mRNA was the lowest (9.9% of that of the control) when the concentration of r-Mt cpn10 was increased to 10 μg/ml (Fig. 3A). Western blot analysis demonstrated a similar trend for the level of OPG protein following treatment with r-Mt cpn10 (0.01, 0.1, 1 and 10 μg/ml) for 8 h, with the level of OPG protein at its lowest when the concentration of r-Mt cpn10 was increased to 10 μg/ml (Fig. 3B and C). These results demonstrated that r-Mt cpn10 inhibited OPG mRNA and protein expression in a dose-dependent manner.

### r-Mt cpn10 upregulates the levels of RANKL mRNA and protein in a time-dependent manner

**Figure 4:**
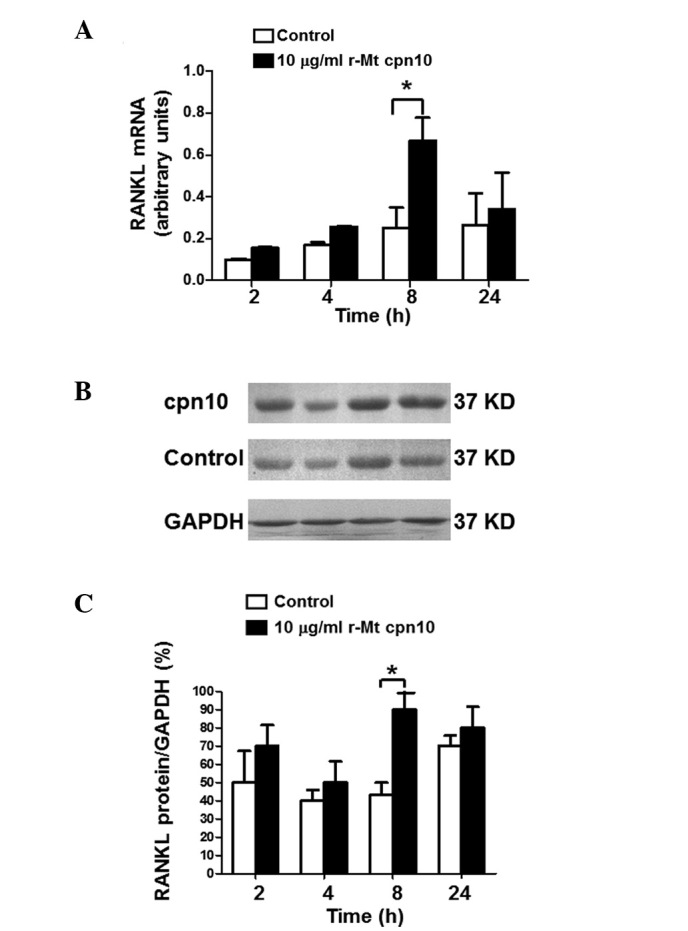
Levels of RANKL in third-generation osteoblasts following treatment with r-Mt cpn10 (10 μg/ml) for 2, 4, 8 and 24 h. (A) Levels of RANKL mRNA following stimulation with control or r-Mt cpn10 (10 μg/ml) for 2, 4, 8 and 24 h. Total RNA was isolated from the harvested cells, transcribed to cDNA and quantified by reverse transcription-quantitative polymerase chain reaction analysis. The amount of RANKL mRNA was compared with the amount of the reference gene (GAPDH) mRNA. Values are expressed as the mean ± intra-assay variation of duplicate analyses. (B) Western blot analysis of RANKL protein expression following treatment with the control or r-Mt cpn10 (10 μg/ml) for 2, 4, 8 and 24 h. (C) Quantification of the level of RANKL protein following treatment with the control or r-Mt cpn10 (10 μg/ml) for 2, 4, 8 and 24 h. Results are expressed as the mean ± standard devation. ^*^P<0.05. RANKL, receptor activator of nuclear factor-κB ligand; r-Mt, recombinant *Mycobacterium tuberculosis*; cpn10, 10-kDa co-chaperonin.

RT-qPCR and western blotting were performed to investigate the time-course effect of r-Mt cpn10 on the level of RANKL. The RT-qPCR results for the third-generation cultured osteoblasts demonstrated that RANKL mRNA expression was induced following treatment with r-Mt cpn10 (10 μg/ml), with a maximal effect of a 2.6-fold induction compared with the control observed after 8 h (Fig. 4A). Similarly, western blot analysis revealed that treatment with r-Mt cpn10 (10 μg/ml) for 8 h increased the level of RANKL protein by 100% compared with the control (Fig. 4B and C). These results suggested that r-Mt cpn10 upregulated the levels of RANKL mRNA and protein in a time-dependent manner.

### Treatment with r-Mt cpn10 increases the RANKL/OPG ratio

**Figure 5:**
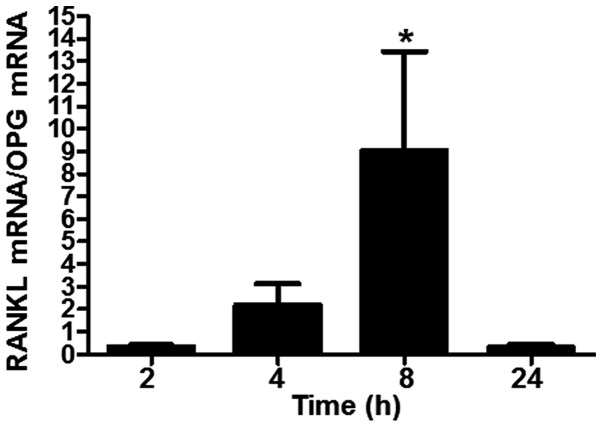
RANKL/OPG ratio following treatment with r-Mt cpn10 (10 μg/ml) for 2, 4, 8 and 24 h. Results are expressed as the mean ± standard devation. ^*^P<0.05 compared with the control. RANKL, receptor activator of nuclear factor-κB ligand; OPG, osteoprotegerin; r-Mt, recombinant *Mycobacterium tuberculosis*; cpn10, 10-kDa co-chaperonin.

To evaluate the net effect of RANKL and OPG in the *in vitro* system, the RANKL/OPG ratio in the third-generation cultured osteoblasts was calculated following treatment with r-Mt cpn10 (10 μg/ml). The results revealed that the RANKL/OPG mRNA ratio was nine-fold higher than that in the control cells. However, after 24 h, the ratio returned almost to its initial value (Fig. 5). This calculation indicated that treatment with r-Mt cpn10 (10 μg/ml) for 8 h had the strongest effect in increasing the RANKL/OPG ratio.

## Discussion

The present study demonstrated that OPG was downregulated and RANKL was upregulated in osteoblasts by r-Mt cpn10. It is well understood that OPG and RANKL are important regulators of osteoclastogenesis, and RANKL has been found to be involved in the differentiation and development of osteoclasts (16). In a previous study, RANKL-knockout (RANKL −/−) mice exhibited extensive bone sclerosis and a lack of mature osteoclasts; this was alleviated following RANKL treatment. Furthermore, the application of recombinant RANKL led to severe osteoporosis and hypercalcemia in mice (17). Transgenic OPG mice (+/+) exhibited bone sclerosis, while OPG-knockout mice (OPG −/−) showed progressive aggravation of osteoporosis, with a reduction in the cortical and trabecular bone volume, thickness and number of cells (18). *In vitro* studies showed that 10 ng/ml recombinant OPG completely inhibited the generation of osteoblasts and osteoclasts in the co-culture system. The addition of RANKL formed fully functional osteoclasts, which were completely inhibited by the addition of OPG (19,20). Thus, the differential regulation of the two proteins may be an important mechanism by which r-Mt cpn10 induces bone resorption.

The relative ratio of RANKL/OPG in the bone marrow microenvironment is the principal factor that defines the effect of cytokines on osteoclast formation and activation. Notably, it has been shown that the serum RANKL/OPG ratio has prognostic significance in multiple myeloma (21). A higher risk of osteolytic lesions and mortality is associated with an increased RANKL/OPG ratio. In previous studies, an increased RANKL/OPG ratio has been observed in patients with severe osteolysis (22–25). In the present study, the levels of RANKL and OPG in osteoblasts were antagonistically affected by r-Mt cpn10, leading to a markedly increased RANKL/OPG ratio. This observation supports our hypothesis that the RANK-RANKL-OPG system mediates r-Mt cpn10-induced bone resorption.

The resorptive effect of r-Mt Cpn10 is partly caused by its direct effect on bone tissue (10). Regions of cpn10 responsible for its osteolytic and osteoblast-antiproliferative activities have been identified to exist in the loop spanning residues 65–70 and the mobile loop of the protein. It has been previously speculated that the most likely signaling pathway occurred via cpn10 receptors (26), with the sequence SGLVIPDT of cpn10 directly binding to RANK. In the present study, the effects of r-Mt cpn10 on OPG and RANKL expression were dose- and time-dependent; however, whether the changes in the levels of mRNA resulted from the regulation of transcription or changes in message stability remains unknown. A reporter assay using RANKL and OPG upstream regulatory sequences is required to confirm the speculation that these are transcriptional effects mediated by cpn10.

In the current study, the effects of r-Mt cpn10 in inducing bone resorption were similar to those reported previously (27–30). The maximal r-Mt cpn10-dependent downregulation of OPG mRNA expression was observed after 4–8 h and that of OPG protein expression after 8 h. The r-Mt cpn10-dependent downregulation of OPG was the most significant at a concentration of 10 μg/ml. The maximal r-Mt cpn10-dependent upregulation of RANKL mRNA and protein expression was observed with 10 μg/ml r-Mt cpn10 after 8 h. Meghji *et al* (13) reported that the addition of Mt to murine calvarial bone produced a dose-dependent stimulation of bone resorption, which was measured from calcium release into the tissue culture medium. The number of osteoclasts in the calvarial explants was counted and the results showed a parallel increase. In the present study, an r-Mt cpn10-dependent downregulation of OPG expression and upregulation of RANKL expression was observed in the presence of 10 μg/ml r-Mt cpn10. The relative ratio of RANKL/OPG was highest at 8 h, and it indicated osteoclast formation and activation.

During the development process, in which the expression levels of OPG and RANKL are regulated, OPG is enhanced during the differentiation of osteoblasts (31), while RANKL expression is reduced as the differentiation progresses. In the osteoblastic cultures used in the current study, OPG expression was higher than RANKL expression. The results suggested that r-Mt cpn10 inhibited OPG mRNA and protein expression in osteoblastic cells at all stages of differentiation.

In conclusion, the present study demonstrated that the effect of r-Mt cpn10 on osteoblastic cells was achieved via an increase in the RANKL/OPG ratio. The paracrine mechanisms by which r-Mt cpn10 induces bone resorption, and thus increases the risk of fractures, may involve a local increase in the level of RANKL and decrease in the level of OPG in the bone microenvironment.
